# Zwitterionic rhodamine-CPT prodrug nanoparticles with GSH/H_2_O_2_ responsiveness for cancer theranostics

**DOI:** 10.7150/thno.78884

**Published:** 2023-01-01

**Authors:** Xueluer Mu, Zejian Huang, Wenbi Feng, Manyu Zhai, Yukun Wang, Dan Zhou, Xianfeng Zhou

**Affiliations:** 1 Key Lab of Biobased Polymer Materials, Shandong Provincial Education Department, College of Polymer Science and Engineering, Qingdao University of Science and Technology, Qingdao, 266042, P.R. China.; 2 Chongqing Key Laboratory of Extraordinary Bond Engineering and Advanced Materials Technology, College of Materials Science and Engineering, Yangtze Normal University, Chongqing, 408100, P.R. China.

**Keywords:** Camptothecin prodrug, zwitterion, disulfide bond, self-assembly, nanomedicine

## Abstract

**Rationale:** Fluorescently traceable prodrugs, which can monitor their biodistribution *in vivo* and track the kinetics of drug delivery in living cells, are promising for constructing theranostic medicines. However, due to their charge and hydrophobicity, most of the fluorescently traceable prodrugs exhibit high protein binding and non-specific tissue retention affecting *in vivo* distribution and toxicity, with high background signals.

**Methods:** Herein, the zwitterionic rhodamine (RhB) and camptothecin (CPT) were bridged with a disulfide bond to construct a tumorous heterogeneity-activatable prodrug (RhB-SS-CPT). The interaction of zwitterionic RhB-SS-CPT with proteins was detected by UV and fluorescence spectroscopy, and further demonstrated by molecular docking studies. Then, intracellular tracking and cytotoxicity of RhB-SS-CPT were determined in tumor and normal cells. Finally, the *in vivo* biodistribution, pharmacokinetics, and anticancer efficacy of RhB-SS-CPT were evaluated in a mouse animal model.

**Results:** The tumorous heterogeneity-activatable RhB-SS-CPT prodrug can self-assemble into stable nanoparticles in water based on its amphiphilic structure. Particularly, the zwitterionic prodrug nanoparticles reduce the non-specific binding to generate a low background signal for better identification of cancerous lesions, achieve rapid internalization into cancer cells, selectively release bioactive CPT as a cytotoxic agent in response to high levels of GSH and H_2_O_2_, and exhibit high fluorescence that contributes to the visual chemotherapy modality. In addition, the RhB-SS-CPT prodrug nanoparticles show longer circulation time and better antitumor activity than free CPT *in vivo*. Interestingly, the zwitterionic nature allows RhB-SS-CPT to be excreted through the renal route, with fewer side effects.

**Conclusions:** Zwitterionic features and responsive linkers are important considerations for constructing potent prodrugs, which provide some useful insights to design the next-generation of theranostic prodrugs for cancer.

## Introduction

For the precise diagnosis and treatment of cancer, prodrugs are typically created by conjugating drugs to molecular probes via stimuli-responsive linkages that permit the release of bioactive drugs in response to endogenous factors in tumor cells 1-7. In the meantime, fluorescence-based probes that can visualize living cells and whole animals hold the promise of real-time, noninvasive, high-resolution imaging for accurate tumor diagnosis. However, most of the fluorescent probes, such as rhodamine 8, cyanine dyes 9, BODIBY 10, coumarin 11, and fluorescein 12, are hydrophobic, or hydrophilic but carry net positive or negative charges. Either of these properties is known to show high protein binding, low serum stability, and yield high non-specific binding *in vivo*, which can mask the signals from cancerous lesions, lowering the imaging resolution to distinguish tumors from normal tissue. Therefore, it is evident that the physicochemical properties of fluorescent probes, including charge density and distribution, hydrophilicity/hydrophobicity, *etc*., can influence the *in vivo* behavior of prodrugs. However, due to the variety of physiological components that exhibit interactions with prodrugs, the *in vivo* fate of fluorescently traceable prodrugs remains poorly understood.

**Figure 1:**
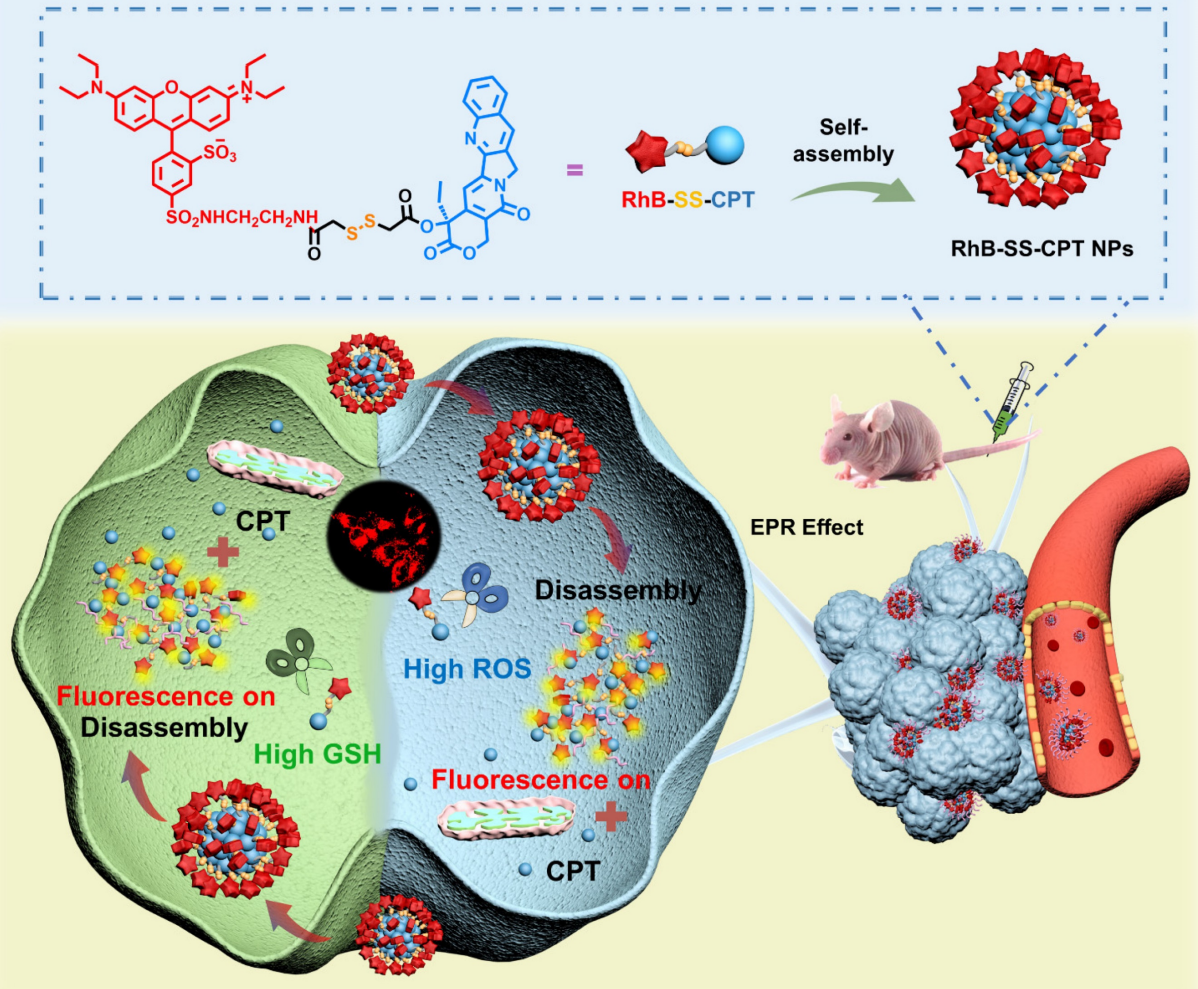
Schematic illustration of disulfide-bridged zwitterionic prodrug nanoparticles, the GSH/H_2_O_2_-responsive drug release and synergistic fluorescence turn-on in tumor cells. The nanoparticles were formed from the zwitterionic RhB-SS-CPT prodrug, which reduces non-specific binding for low background signals.

Previous studies have demonstrated that electrostatic and hydrophobic interactions are the main driving force for non-specific binding 13. Thus, electrostatically neutral hydrophilic polymers such as poly(ethylene glycol) (PEG) 14, 15 or poly(zwitterion) (PZW) 16-18 are widely used as drug carriers, which can interact with water via dipole-dipole or charge-dipole interactions to inhibit non-specific binding. However, polymeric formulations present far more complex problems regarding pharmaceutical-scale production, drug loading ratio, and the potential long-term toxicity of the polymeric carriers 19. Recently, hydrophilic zwitterionic fluorescent probes, mimicking the cell membrane, were designed to achieve low serum binding and non-specific tissue retention 20, 21. Based on this strategy, we fused the hydrophilic zwitterionic sulforhodamine derivative as the fluorescent probe with the hydrophobic anticancer drug camptothecin (CPT), testing the hypothesis that the resulting zwitterionic prodrug (RhB-SS-CPT) would reduce the extent of non-specific binding to proteins or tissues (Figure 1). Sulforhodamine derivatives are an excellent class of fluorophores due to their sufficiently high photostability and brightness, and they exist in dynamic equilibrium between fluorescent zwitterion and non-fluorescent but cell-permeable spirolactone 22-24. The single isomer with the sulfonamide in the para-position (Figure 1) was utilized in the construction of rhodamine-based prodrugs due to its zwitterionic properties and physiological pH-insensitive fluorescence. In light of over-produced glutathione (GSH) and hydrogen peroxide (H_2_O_2_), the redox-responsive disulfide bond was used to link RhB with CPT 3, 25-28, with high sensitivity to the disparate pathophysiological feature (intertumoral heterogeneity). RhB-SS-CPT prodrug spontaneously self-assembled in an aqueous physiological environment due to its inherent amphiphilic structure. Hydrophobic interactions, π-π stacking, and hydrogen bonds, among others, contributed to the enhanced stability of the formed nanoparticles in RhB-SS-CPT. Notably, the hydrophilic zwitterionic charges could be distributed uniformly across the nanoparticles to shield the relatively large hydrophobic CPT. Ultimately, the zwitterionic RhB-SS-CPT nanoparticles visualize their tissue and cellular distribution in real-time and enable the rapid CPT release in cancer cells to suppress tumor growth spatially and temporally. Our findings offer novel insights into the rational design of prodrugs, specifically responsiveness and zwitterionicity, which may offer a promising solution for cancer theranostics.

## Experimental section

### Synthesis of RhB-SS-CPT

The CPT-SS-COOH (100 mg, 0.19 mmol), compound 5 (129 mg, 0.22 mmol), and HBTU (148 mg, 0.39 mmol) were dissolved in dichloromethane (25 mL) at 0 °C in nitrogen atmosphere. After stirred for 0.5 h, DIPEA (50 mg, 0.39 mmol) was added dropwise. The reaction was continued at room temperature for 5 hours, then washed with 5% HCl (10 mL), water (20 mL), and dried over NaSO_4_. The organic phase was removed, and the crude product was separated and purified by silica gel column chromatography using CH_2_Cl_2_:CH_3_OH (50:1, v/v) as eluent to give a red solid as RhB-SS-CPT. Yield: 29.3%. ^1^H NMR (400 MHz, CDCl_3_) δ (ppm): 8.67 (s, 1H), 8.32 (s, 1H), 8.11 (d, *J* = 8.4 Hz, 1H), 7.89 (d, *J* = 8.8 Hz, 1H), 7.78 (d, *J* = 8 Hz, 1H), 7.69 (t, *J* = 6.8 Hz, 1H), 7.49 (t, *J* = 4.4 Hz, 1H), 7.31 (s, 1H), 7.22-7.16 (m, 2H), 6.84 (m, 3H), 6.58 (s, 2H), 5.34 (m, 2H), 5.12 (s, 2H), 3.47 (m, 12H), 3.17 (s, 2H), 3.04 (s, 2H), 2.13 (m, 2H), 1.22 (t, *J* = 12.4 Hz, 12H), 0.93 (t, *J* = 7.2 Hz, 3H). ^13^C NMR (100 MHz, CDCl_3_): δ (ppm): 169.32, 168.98, 167.45, 157.68, 157.26, 155.59, 146.81, 145.32, 141.76, 133.78, 133.52, 128.51, 128.21, 128.14, 127.93, 127.65, 120.11, 114.30, 114.25, 114.09, 113.98, 96.52, 95.51, 66.36, 50.12, 45.89, 42.51, 42.13, 40.48, 39.49, 31.71, 31.44, 30.20, 29.69, 22.56, 14.16, 12.66, 7.63. MS: calculated for C_53_H_54_N_6_O_12_S_4_ (M): 1095.285; found: 1095.168 (M).

### Molecular Docking

The crystal structure of BSA (PDB entry: 4f5s) was retrieved from RSCB database (https://www.rcsb.org/) and then prepared by AutoDock 1.5.6, including deletion of ligand and addition of charges. The structure of zwitterion RhB is optimized using GaussView 6.0 with DFT method. We used AutoDock 1.5.6 and Pymol to construct the 3D models of BSA and zwitterion RhB, and simulate the 1:1 bound conformation. The docking results yielded the 30 most-trusted ligand configurations, and the interactions between RhB-BSA were given by Discovery Studio 2016.

### *In vivo* anticancer efficacy

All animal experiments were approved by the Department of Science and Technology of Shandong Province and the Laboratory Animal Center of Qingdao Hao Biological Engineering Co., Ltd.

4T1 cells (3 × 10^6^ cells per 200 µL) were injected subcutaneously into the right hind leg of female nude mice (6 weeks old, ~ 15 g). When the tumor volume reached approximately 100 mm^3^, mice were intravenously administrated with free CPT or RhB-SS-CPT nanoparticles at a dose of 5 mg/kg equivalent to CPT (n = 5 for each group). Saline was used as control. The free CPT or RhB-SS-CPT nanoparticles were injected every three days for 20 days. The mice were sacrificed after 21 days of treatment, and the heart, liver, spleen, lung, kidney and tumors were collected for hematoxylin and eosin (H&E) staining and analysis.

### Statistical analysis

Data were presented as mean ± SEM. Comparisons were made by using one-way ANOVA with post-hoc testing or Student's t-test. *P*-values of <0.05 were considered statistically significant.

## Results and Discussion

### Design and synthesis of RhB-SS-CPT prodrug

Scheme S1 depicts the synthesis of RhB-SS-CPT prodrug in the supporting information. The structures of intermediates were confirmed by ^1^H NMR (Figure S2-S3), and the final prodrug RhB-SS-CPT was confirmed by ^1^H NMR, ^13^C NMR, and mass spectrometry (MALDI-TOF, Figure S4-S6). According to previous literature, the key intermediate compound 4 exists in two isomers. The isomer 4', with the sulforhodamine ortho to the xanthylium ring system, is capable of spirocyclization, and exists in dynamic equilibrium between fluorescent zwitterion and non-fluorescent spirolactone 22, 24. Since the isomer 4 is homogeneous under physiological conditions and is unaffected by pH-induced changes in fluorescence, it was well separated and conjugated with CPT to create the RhB-SS-CPT prodrug, which was based on the hypothesis that zwitterionic RhB might enable prodrug with low serum binding and ultralow non-specific tissue background (Figure S7).

### Self-assembly of RhB-SS-CPT prodrug

**Figure 2:**
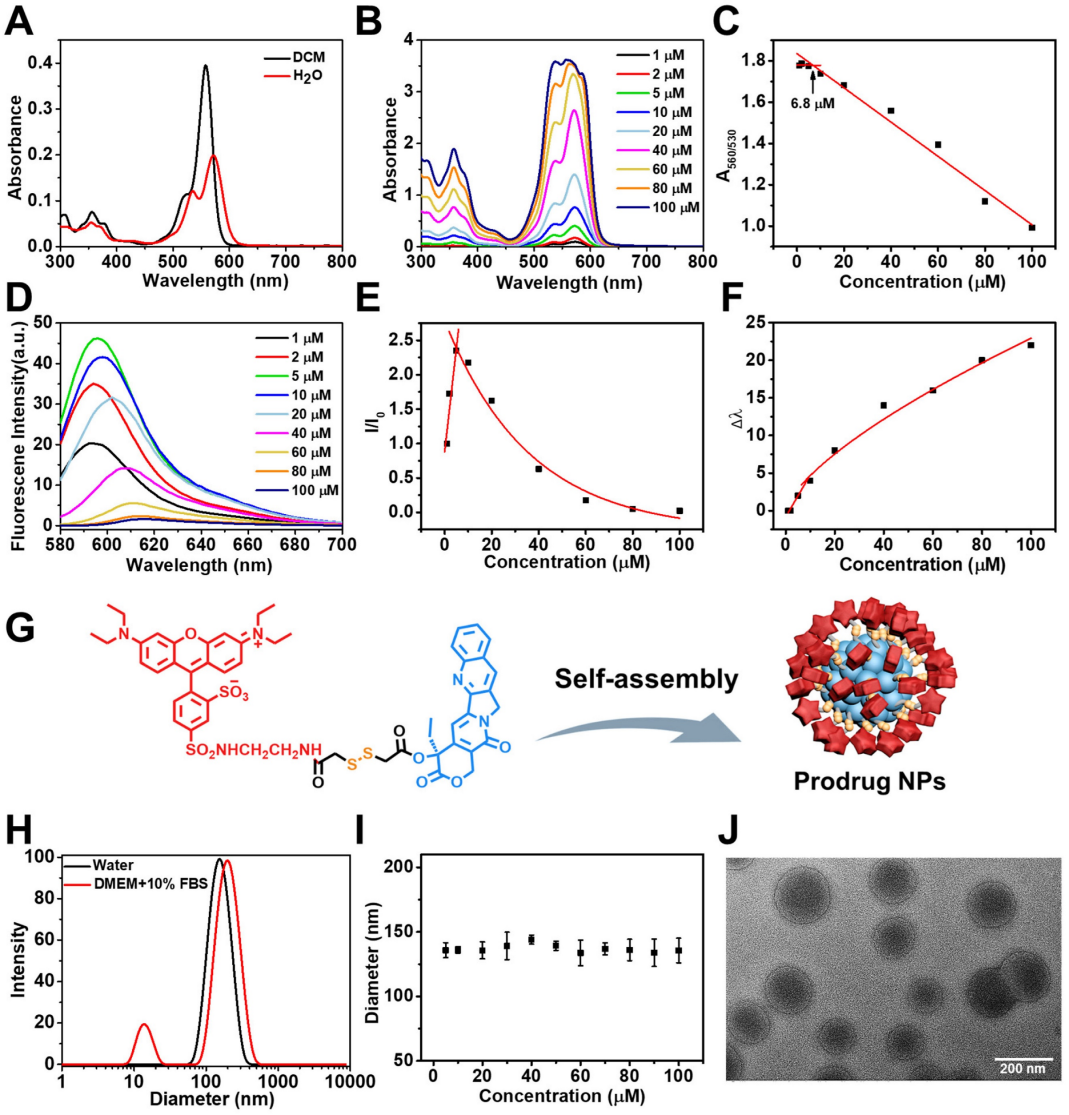
** (A)** Absorption spectra of RhB-SS-CPT (5 μM) in dichloromethane and water. **(B)** Concentration-dependent absorption spectra of RhB-SS-CPT in water. **(C)** Changes in the ratio of the absorption intensities at 530 and 560 nm with the different concentrations of RhB-SS-CPT. **(D)** Concentration-dependent fluorescence spectra of RhB-SS-CPT in water. **(E)** Normalized changes in fluorescence of RhB-SS-CPT with different concentrations in water. **(F)** Stokes shifts are plotted as a function of RhB-SS-CPT concentration in water. **(G)** Schematic representation of the formation of RhB-SS-CPT prodrug nanoparticles by self-assembly with the hydrophobic CPT forming the core and the hydrophilic RhB zwitterion at the exterior. **(H)** DLS size profile of RhB-SS-CPT nanoparticles in water or DMEM supplemented with 10% FBS. **(I)** Particle size distribution of RhB-SS-CPT in water with different concentrations. Error bars show ± s.d. from 3 experiments. **(J)** TEM images of RhB-SS-CPT nanoparticles recorded in water.

The prodrug RhB-SS-CPT is soluble in organic solvents. UV-Vis absorption and fluorescence spectroscopy were used to determine the optical properties of the RhB-SS-CPT prodrug. As shown in Figure 2A, the absorption spectrum of RhB-SS-CPT in organic solvent consists of a peak at ~ 560 nm and a small shoulder at ~ 530 nm. In an aqueous solution, the peak absorption at ~ 560 nm decreases and is slightly bathochromic shifted. The shoulder at ~ 530 nm increases relative to the ~ 560 nm peak, which can be attributed to the formation of the H-dimer of RhB 29. Increasing the concentration of RhB-SS-CPT prodrug from 1 µM to 100 µM in water (Figure 2B), the ratio of A_560_/A_530_ nm initially levels off and then decreases from a minimum concentration of ~ 6.8 µM (Figure 2C), which is suggested as its critical micelle concentration (CMC) in water. In addition, the maximum emission intensity of RhB-SS-CPT prodrug in water was increased at lower concentrations as its concentrations increased (Figure 2D). As concentrations increased beyond 5 µM, the peak maxima shifted to lower energies, and intensities decreased (Figure 2E & 2F). Based on aggregation-caused quenching (ACQ), the lower emission intensity of RhB-SS-CPT in water is due to the presence of aggregates. Consequently, these optical properties of RhB-SS-CPT prodrug with amphiphilic structure indicate that RhB-SS-CPT can self-assemble to form nanoparticles in an aqueous solution via a straightforward, rapid injection nanoprecipitation method. The hydrophobic CPT could form the core, whereas the hydrophilic RhB zwitterion surrounded it (Figure 2G). Figure 2H demonstrates that the RhB-SS-CPT prodrug nanoparticles have a mean hydrodynamic size of approximately 144 nm (PDI = 0.091; DLS). Notably, the radius of RhB-SS-CPT nanoparticles is independent of concentration (Figure 2I), which is attributable to the nanoparticles' energetic stabilization by zwitterions. In addition, transmission electron microscope (TEM) reveals that RhB-SS-CPT prodrug aggregates in water are approximately 130 nm in diameter nanoparticles (Figure 2J). The size of RhB-SS-CPT nanoparticles makes them suitable for targeting the tumor *in vivo*, based on the enhanced permeability and retention (EPR) effect.

### Protein absorption of zwitterionic RhB-SS-CPT nanoparticles in serum

**Figure 3:**
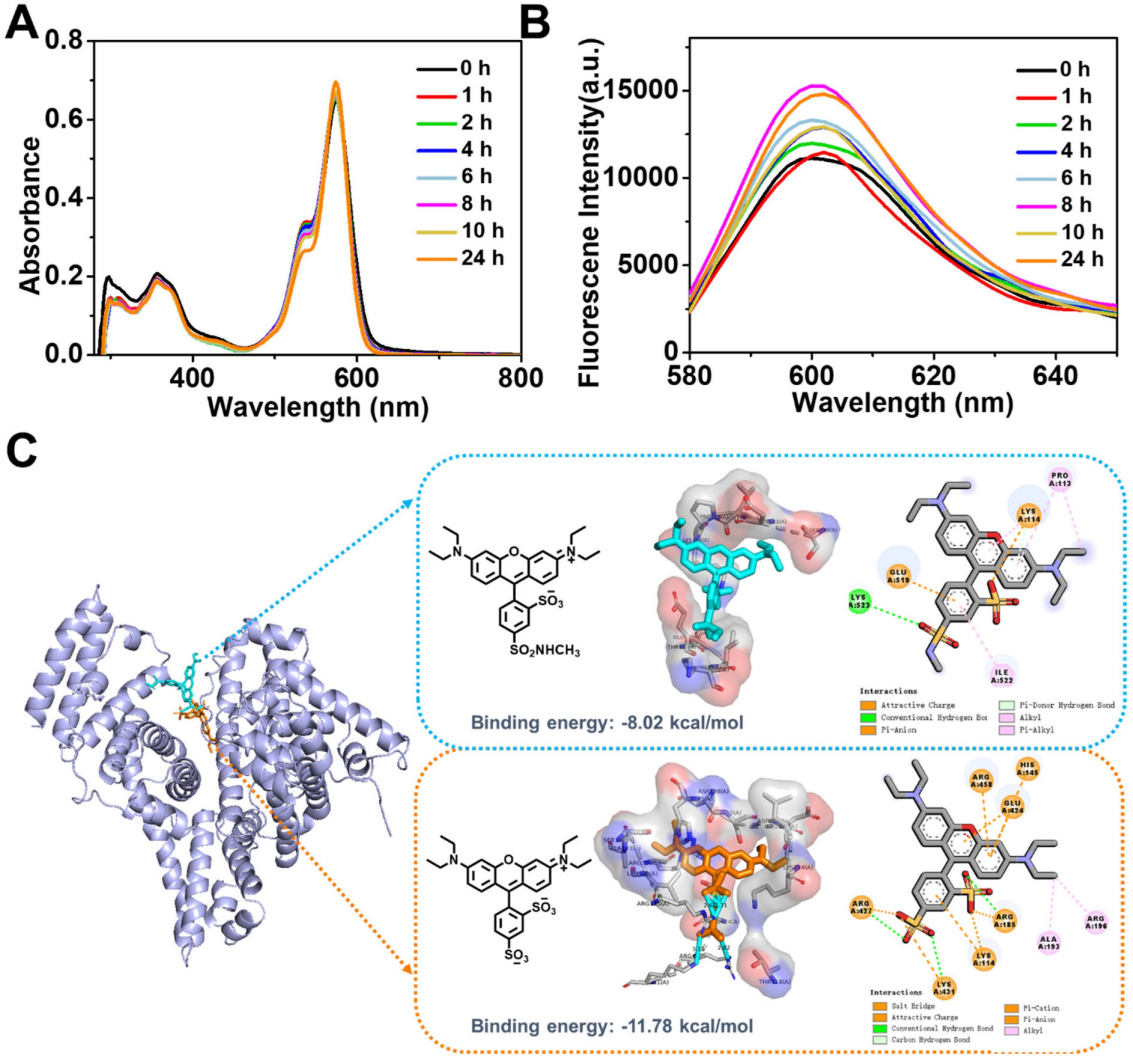
Time-dependent absorption** (A)** and fluorescence **(B)** spectra of RhB-SS-CPT (5 μM) in DMEM supplemented with 10% FBS. **(C)** Predicted structure of the zwitterionic RhB or sulforhodamine B bound to BSA using molecular docking technique.

Our initial expectation was that the zwitterionic probe provided a key structure for the self-assembled nanoparticles to exhibit low protein binding and improved stability in the presence of serum. We characterized the RhB-SS-CPT prodrug in cell culture media (with 10% FBS) using UV-Vis absorption and fluorescence spectroscopy (Figure 3A & B). Absorption and fluorescence spectroscopy are both high-resolution techniques that we consider the gold standard for determining the non-specific binding that may affect the stability of RhB-SS-CPT nanoparticles in cell culture media. Previously, sulforhodamine B displayed increased fluorescence with a net charge of -1 bound to serum albumin, resulting in a stronger background signal 30. Comparative data from time-based absorption and fluorescence spectra of RhB-SS-CPT indicated that biological media have little effect on the spectra of prodrug nanoparticles and that their nanostructures were comparable to those of pure nanoparticles. RhB-SS-CPT nanoparticles exhibited only a modest increase in hydrodynamic diameter, and there was no sign of precipitation in the physiological medium (Figure 2H). The stability of RhB-SS-CPT nanoparticles in serum albumin-containing cell culture medium was investigated further. According to the DLS results, RhB-SS-CPT nanoparticles were stable within 48 h (Figure S8). The CMC of RhB-SS-CPT in FBS-supplemented cell medium was ~ 4.9 µM, a slight increase over its CMC in water (~ 3.9 µM, by DLS), suggesting a weak interaction of the albumin with RhB-SS-CPT (Figure S9). The optimized conformation of sulforhodamine B or zwitterionic RhB was used in a molecular docking study to obtain more information regarding the binding sites between RhB and albumins, such as bovine serum albumin (BSA). For sulforhodamine B, the binding energy was determined to be -11.78 kcal/mol, indicating a strong bind affinity of sulforhodamine B to BSA (Figure 3C). At least seven charged amino acid residues such as LYS 114 (A), HIS 145 (A), ARG 185 (A), GLU 424 (A), ARG 427 (A), LYS 431 (A), and ARG 458 (A), form an interface pocket surrounding sulforhodamine B, indicating that electrostatic interaction is one of the main driving forces for the binding of sulforhodamine B to BSA. The enhanced fluorescence intensity of sulforhodamine B is directly related to BSA binding, which hampers its TICT process to restrict thermal rotation. As a comparison, Figure 3C also shows the most probable binding site for zwitterionic RhB in BSA with the binding energy at -8.02 kcal/mol, which suggests that zwitterionic RhB binds at a shallower position in BSA with a weaker binding affinity. A few amino acid residues, including PRO113 (A), LYS 114 (A), GLU 519 (A), LYS 523 (A), and ILE 522 (A), are responsible for binding via electrostatic force or stacking. Albumin and RhB-SS-CPT nanoparticle interactions may be dynamic, acting as a “soft” protein corona that reduces the non-specific binding of RhB-SS-CPT nanoparticles and contributes to their long blood circulation. Collectively, these findings demonstrated that the zwitterionic RhB on the surface of RhB-SS-CPT nanoparticles could exhibit a low background signal due to its resistance to protein absorption.

### Redox-responsive CPT release and release mechanism

**Figure 4:**
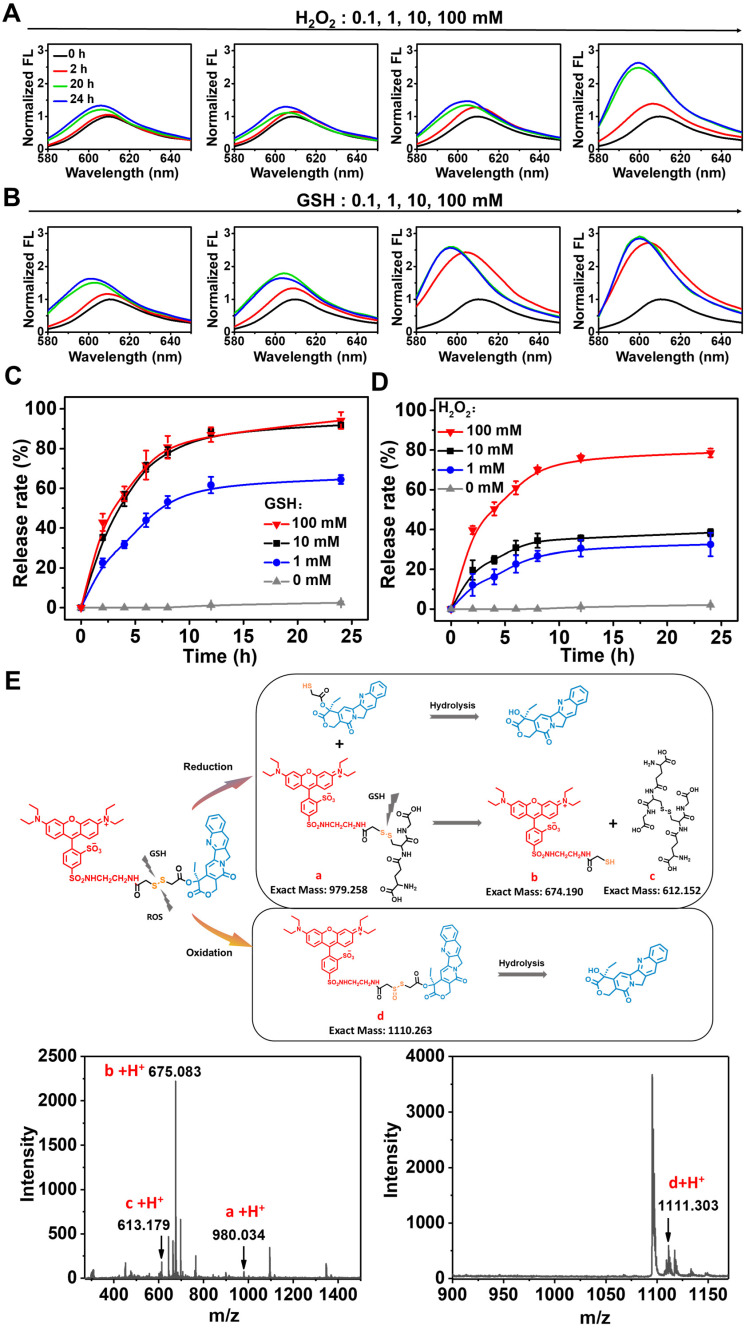
Time-dependent fluorescence changes of RhB-SS-CPT prodrug (50 µM) in the presence of different concentrations of H_2_O_2_ **(A)** or GSH **(B)**. CPT release from RhB-SS-CPT prodrug nanoparticles (100 µM) as a function of time in the presence of different concentrations of GSH **(C)** or H_2_O_2_ **(D)**. CPT was detected by UV absorption in HPLC. Error bars show ± s.d. from 3 experiments. **(E)** The proposed mechanism of GSH- and H_2_O_2_-mediated CPT release from RhB-SS-CPT prodrugs (50 µM) was derived from the ESI-MS analysis of RhB-SS-CPT treated with the corresponding stimuli for 6 h.

To restore the therapeutic efficacy of the prodrug, it must be efficiently converted to its active form. Previously, it was believed that disulfide bonds were sensitive to both reduction and oxidation stimuli 31-33. Compared to normal cells, tumor cells produce more H_2_O_2_ and GSH simultaneously, allowing for intracellular drug release on demand. Fluorescence spectra of RhB-SS-CPT upon reactions with GSH or H_2_O_2_ were analyzed to determine the active drug release behavior of the prodrug containing a disulfide bond. At H_2_O_2_ concentrations above 100 mM (Figure 4A) or GSH concentrations above 10 mM (Figure 4B), the time-dependent rapid fluorescence enhancement of RhB-SS-CPT prodrug was observed, indicating that CPT was released from the prodrug nanoparticles in a concentration-dependent manner. Using high-performance liquid chromatography, we investigated the release behavior of active CPT (HPLC) quantitatively. The CPT release kinetics are presented in Figure 4C and 4D. About 80% of CPT was released from prodrug nanoparticles in the presence of 10 mM of GSH within 12 h, while less than 30% of CPT was released in the presence of 10 mM of H_2_O_2_ over the same period. As the concentration of H_2_O_2_ increased to 100 mM, the RhB-SS-CPT prodrugs continuously released CPT, and over 75% of CPT was released after 12 h of incubation. In addition, we evaluated the release of CPT from RhB-SS-CPT nanoparticles in the presence of lower concentrations of H_2_O_2_ or GSH, and RhB-SS-CPT exhibited faster reduction responsiveness than oxidation responsiveness to release active CPT. To understand the mechanism, we incubated prodrug nanoparticles with 10 mM of GSH or H_2_O_2_ and investigated its change of molecular weight by using electrospray ionization mass spectrometry (ESI-MS). The mass spectra of RhB-SS-CPT treated with GSH showed a molecular weight of 980.034 [RhB+GSH, **a**], 675.083 [RhB-SH, **b**], and 613.179 [GSSG, **c**], suggesting that a disulfide bond- thiol bond exchange reaction occurred (Figure 4E). This reaction occurred rapidly, and the resulting hydrophilic thiol could facilitate the hydrolysis of adjacent ester bonds to liberate active CPT 31. As a comparison, the mass spectra of RhB-SS-CPT treated with H_2_O_2_ exhibited molecular peaks of 1095.344 [RhB-SS-CPT] and 1111.303 [RhB-SS-CPT+monoxide, **d**], indicating the formation of hydrophilic sulfoxide that could also facilitate the hydrolysis of the adjacent ester bond. Notably, a significant quantity of unreacted RhB-SS-CPT was still present, indicating that H_2_O_2_ oxidation is a slow process. These findings indicate that the redox microenvironment of tumor cells may trigger significantly greater and more rapid CPT release from prodrug nanoparticles than under normal physiological conditions.

### Intracellular tracking and cytotoxicity

**Figure 5:**
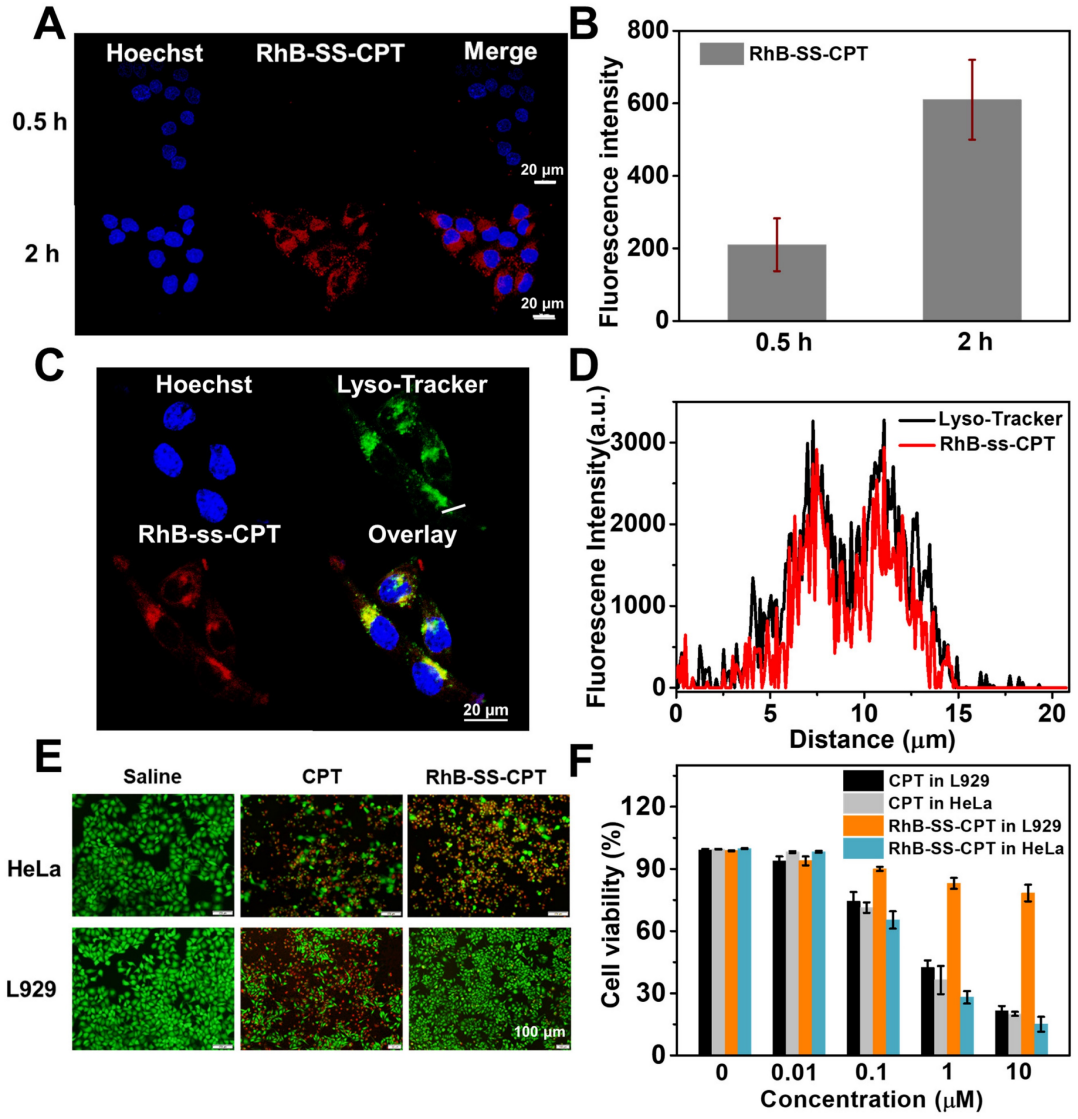
** (A)** Confocal microscopy images of RhB-SS-CPT prodrug nanoparticles in HeLa cells after 0.5 or 2 h incubation. The scale bar is 20 µm. **(B)** Quantitative analysis of fluorescence intensity in HeLa cells in (A). The error bars show the mean ± s.d. of 5 different positions. **(C)** Confocal microscopy images of RhB-SS-CPT prodrug nanoparticles in HeLa cells costained with LysoTracker Green^®^. The scale bar is 20 µm. **(D)** The intensity profile of ROI lines. **(E)** Live/Dead fluorescent staining of HeLa cancer cells or L929 normal cells incubated with CPT or RhB-SS-CPT (10 μM) for 72 h. Saline was used as a control. **(F)** Cytotoxicity of different concentrations of CPT or RhB-SS-CPT in HeLa cancer cells and L929 normal cells was evaluated by MTT assay. Error bars show ± s.d. from 3 experiments.

We further investigated the uptake of RhB-SS-CPT nanoparticles by cancer cells using confocal laser scanning microscopy (CLSM). The zwitterionic RhB-SS-CPT nanoparticles showed much lower cellular uptake in the first 30 min compared to the charged sulforhodamine B (Figure 5A & B). The relatively slow uptake kinetics could be attributed to the zwitterionic surface of the RhB-SS-CPT nanoparticles, whose neutral zeta potential reduces protein binding to cell surfaces (Figure S10). RhB-SS-CPT nanoparticles entered the cells and accumulated in the cytoplasm, as evidenced by the minimal co-localization of RhB's red fluorescence with blue emissions from the nuclei-specific probe Hoechst 33342, as seen in the cell imaging. RhB-SS-CPT nanoparticle subcellular localization was determined by co-staining cells with RhB-SS-CPT nanoparticles and the commercially available lysosome-specific staining probe LysoTracker Green^®^. The yellow fluorescence in Figure 5C indicated the same subcellular localization of LysoTracker Green® with RhB-SS-CPT nanoparticles. The co-localization percentage was quantified using Pearson's sample correlation factors (Rr) with a value of 77.3 ± 3.5%, indicating a high subcellular distribution of RhB-SS-CPT nanoparticles in lysosomes (Figure 5D). Furthermore, cytotoxicity of RhB-SS-CPT on HeLa cancer cells and normal L929 cells was evaluated through Live/Dead staining and a standard MTT assay. When the concentration of CPT was higher than 1 µM, the cell viability of free CPT was less than 35% in both HeLa and L929 cells (Figure 5E & F). RhB-SS-CPT nanoparticles exhibited a comparable therapeutic effect to free CPT in HeLa cells but exhibited lower cytotoxicity in L929 cells (Figure 5E & F), which was attributed to the different redox environments of cancerous and normal cells. This accords with earlier observations in the buffer (Figure 4). These results suggested that RhB-SS-CPT nanoparticles selectively eliminated cancer cells *in vitro*.

### *In vivo* distribution and pharmacokinetic study

The *in vivo* distribution of RhB-SS-CPT nanoparticles in 4T1 tumor-bearing nude mice was investigated. At predetermined time intervals, mice were sacrificed, and their heart, liver, spleen, lung, kidneys, and tumors were harvested for fluorescence signals analysis. As shown in Figure 5A, 6 h after injection, RhB-SS-CPT nanoparticles passively accumulated in tumor tissue efficiently via the EPR effect, generating a stronger fluorescent signal in the tumor than in the liver, the predominant metabolic organ for drugs. The highest fluorescence intensity (4.9 ± 0.8 ×10^8^ p/sec/cm^2^/sr) in tumor was achieved at 12 h after injection. The backgrounds in nontumor tissues such as heart (0.45 ± 0.07 ×10^8^ p/sec/cm^2^/sr), spleen (0.88 ± 0.16 ×10^8^ p/sec/cm^2^/sr), and lung (1.4 ± 0.4 ×10^8^ p/sec/cm^2^/sr) were much lower, indicating a higher selectivity for RhB-SS-CPT in tumor (Figure 5B). After 12 h, RhB fluorescence gradually disappeared from the tumors. Notably, RhB-SS-CPT still exhibited a high background in the liver, indicating that at least a portion was eliminated from circulation via the hepatobiliary pathway. This could be contrary to the previously reported *in vivo* clearance mechanism of zwitterionic dyes by the kidney 20, 21, possibly due to the exposed CPT residue recognized by the liver. The fluorescence ratio of tumor to kidney decreased from 2.62 to 0.79 within 48 h, which could be related to renal clearance. The blood decay kinetics of RhB-SS-CPT was also studied. Free CPT was cleared very quickly, with a half-life of several minutes. In contrast, the half-time of RhB-SS-CPT was ~4.4 h, indicating prolonged blood circulation (Figure S11). These results suggested that the zwitterionic RhB-SS-CPT nanoparticles were able to accumulate at tumor sites with reduced non-specific tissue uptake and increased renal clearance.

### *In vivo* anticancer efficacy

**Figure 7:**
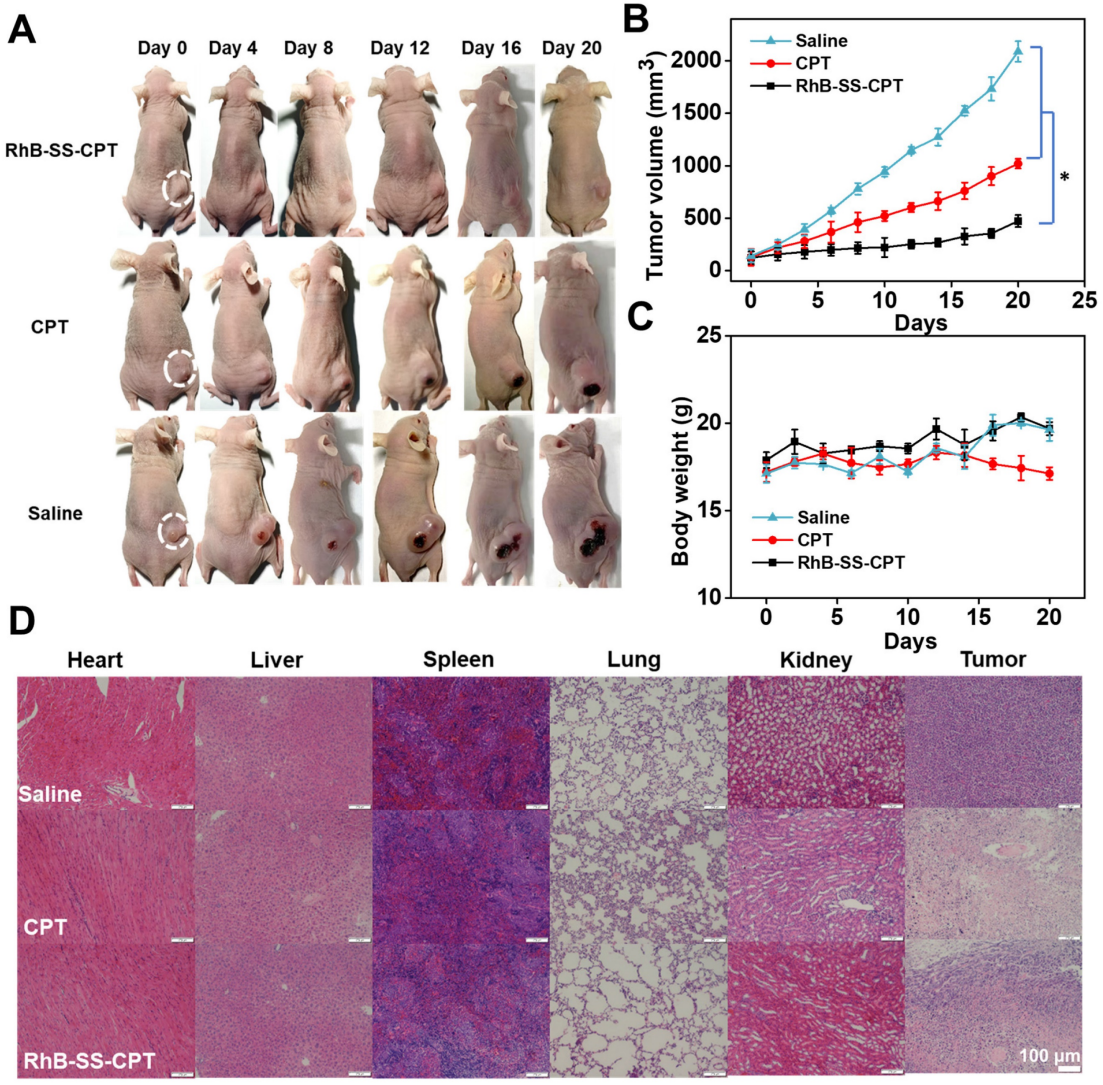
Anticancer efficacy of RhB-SS-CPT prodrug i.v. administered at a CPT-equivalent dose of 5 mg/kg every 3 days. **(A)** Representative photographs of 4T1 cell-bearing mice treated with saline, free CPT, or RhB-SS-CPT nanoparticles for 21 days. Tumor locations are shown with ellipses. **(B)** Tumor volume and** (C)** body weight of treated mice. Error bars in (B) & (C) represent mean ± SEM, n = 5. *P < 0.05. **(D)** Pathological examination of organs or tumors resected from the treated mice.

Encouraged by the excellent properties of RhB-SS-CPT nanoparticles, we further evaluated them *in vivo* anticancer efficacy on 4T1 tumor-bearing nude mice by intravenously injecting RhB-SS-CPT or CPT at CPT equivalent dose of 5 mg/kg. The mice treated with saline were used as a negative control. As shown in Figure 7A, both RhB-SS-CPT nanoparticles and free CPT exhibited potent anticancer effects to inhibit the growth of 4T1 tumors. On day 21, the tumor inhibition of RhB-SS-CPT nanoparticles was ~77.4%, which was 1.5 times that of the free CPT group (~51.2%), implying its better tumor inhibition ability that could be attributed to the longer circulation time and passive tumor targeting ability of RhB-SS-CPT nanoparticles (Figure 7B). The body weight of mice was also monitored, and only the weight in the free CPT group was slightly reduced (Figure 7C), indicating that the side effects of treatment with RhB-SS-CPT were negligible. A blood sample of each mouse in the “saline”, “CPT” and “RhB-SS-CPT” groups was collected, which was tested by blood chemistry indices to show that all of the values of the “RhB-SS-CPT” group were within the range of reference (Figure S12). In addition, in mice, H&E-stained tissue sections of major organs such as the heart, liver, spleen, lung, and kidney revealed no obvious histological damage (Figure 7D), further demonstrating the safety of RhB-SS-CPT treatment. Notably, the RhB-SS-CPT-treated tumor section exhibited more apoptotic cells and lower tumor cellularity than the untreated tumor section (Figure S13). These results confirmed the anticancer efficacy and safety of the zwitterionic RhB-SS-CPT nanoparticles.

## Conclusions

In conclusion, we have developed a zwitterionic activatable theranostic prodrug platform (RhB-SS-CPT) that can track drug delivery kinetics and monitor *in vivo* distribution. RhB-SS-CPT can self-assemble into nanoparticles that, upon redox-induced disulfide cleavage in the tumor microenvironment, release the active CPT and activate fluorescence. RhB-SS-CPT nanoparticles are most notable for their ability to effectively reduce non-specific binding to generate a low background signal for improved identification of cancerous lesions. RhB-SS-CPT nanoparticles exhibit significantly enhanced anticancer efficacy *in vivo* with fewer adverse effects than free CPT. Overall, these results demonstrate that zwitterionic RhB-SS-CPT is a promising self-delivering and tumor-activatable prodrug, and they provide valuable insight for designing the next generation of theranostic prodrugs for cancer.

## Supplementary Material

Supplementary figures, materials and methods.

## Figures and Tables

**Figure 6:**
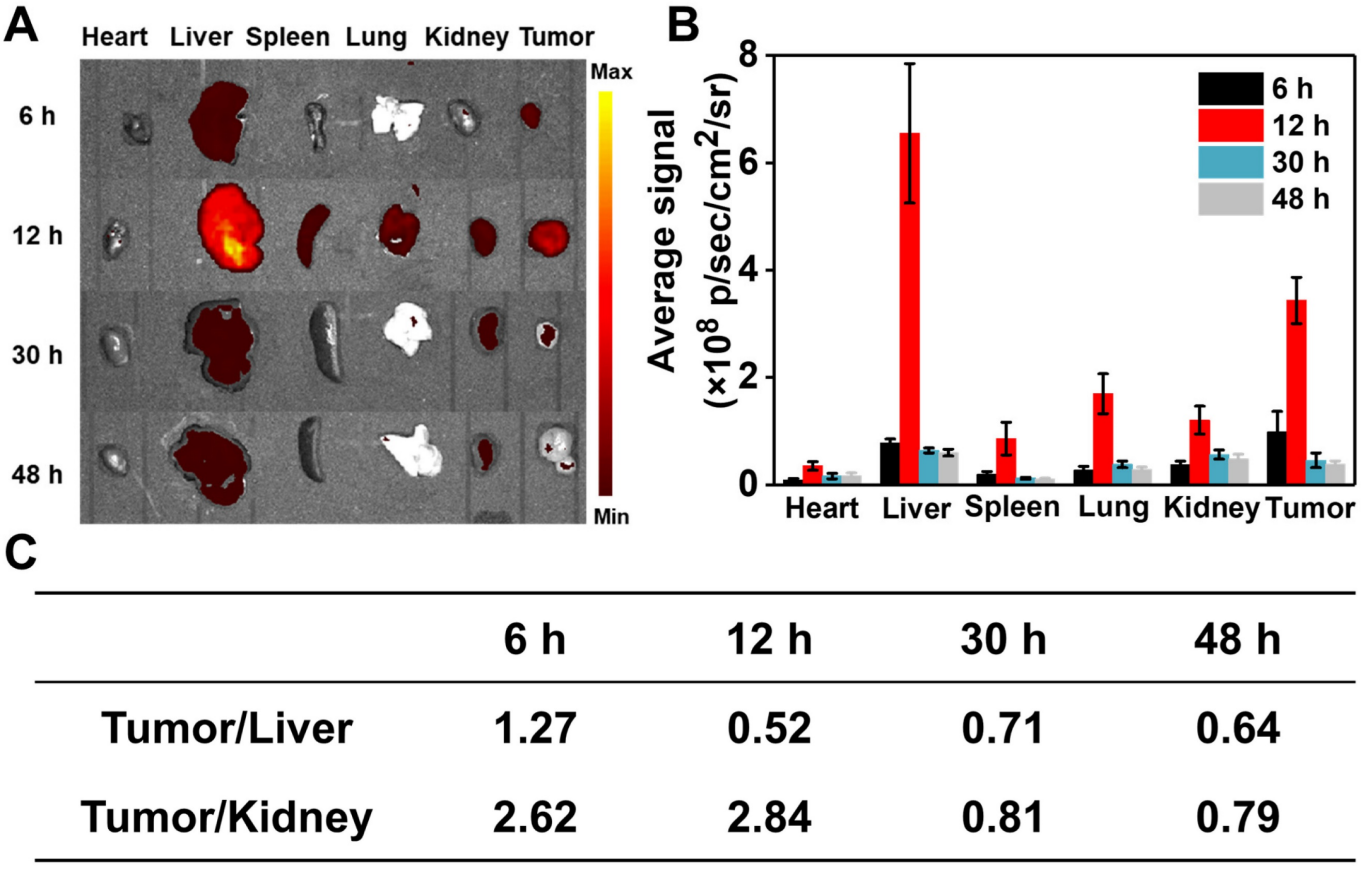
Biodistribution of RhB-SS-CPT prodrug nanoparticles. **(A)** Fluorescent imaging of heart, liver, spleen, lung, kidney, and tumor of mice 6 h, 12 h, 30 h, and 48 h after intravenous injection of RhB-SS-CPT prodrug.** (B)** Quantitative results of RhB-SS-CPT prodrug distribution in different organs and tumors at different times. Error bars are ± s.d. for 3 experimental animals. **(C)** The fluorescent ratios of tumor/liver and tumor/kidney at different times.

## Abbreviations

RhB: rhodamine
CPT: camptothecin
GSH: glutathione
CMC: critical micelle concentration
BSA: bovine serum albumin
