# Supplementary material for: Zwitterionic rhodamine-CPT prodrug nanoparticles with GSH/H2O2 responsiveness for cancer theranostics
Source: Theranostics. 2023 Jan 1;13(1):267–77. doi: 10.7150/thno.78884 (PMC9800722; doi:10.7150/thno.78884)
Supplement: Supplementary file 1 — Supplementary figures, materials and methods. [file thnov13p0267s1.pdf]

Supporting Information

Zwitterionic Rhodamine-CPT Prodrug Nanoparticles with GSH/H_2O_2 Responsivity for Cancer Theranostics

Xueluer Mu [a,#], *Zejian Huang* [a,#], *Wenbi Feng* [a], *Manyu Zhai* [a], *Yukun Wang* [a], *Dan Zhou* [b], *and Xianfeng Zhou* [a,*].

[a.] Key Lab of Biobased Polymer Materials, Shandong Provincial Education Department, College of Polymer Science and Engineering, Qingdao University of Science and Technology, Qingdao, 266042, P.R. China.

[b.] Chongqing Key Laboratory of Extraordinary Bond Engineering and Advanced Materials Technology, College of Materials Science and Engineering, Yangtze Normal University, Chongqing, 408100, P.R. China.

[*] To whom correspondence should be addressed.

E-mail: xianfeng@qust.edu.cn. Phone: +86-532-84022950

Materials and instruments

Camptothecin (CPT), 4-Dimethylaminopyridine (DMAP), N-Boc-ethylenediamine, Trifluoroacetic acid, L-Glutathione reduced (GSH), and 2-(1H-Benzotriazole-1-yl)-1,1,3,3-tetramethyluronium hexafluorophosphate (HBTU) were purchased from Energy Chemical (Shanghai, China). Oxalyl chloride was obtained from Macklin (Shanghai, China). Sulforhodamine B sodium salt was purchased from Aladdin (Shanghai, China). Dithiodiglycolic Acid was obtained from BidePharmatech. Ltd (Shanghai, China). Hoechst 33342, MitoTracker® Green FM, LysoTracker Green® DND-26, Calcein (AM) and ethidium homodimer-1 (EthD-1) were purchased from Invitrogen (Carlsbad, CA). All other reagents and solvents were used without further purification.

^{1}H and ^{13}C nuclear magnetic resonance spectra were recorded using a Bruker Ascend 400 FT-NMR. Mass analysis was measured at Brooke MicroFlex LRF in positive ion mode. Malvern Zetasizer Nano ZS90 was used to measure the particle size distribution at room temperature. HITACHI U-2910 UV-Vis absorption spectrometer and Hitachi F-2700 spectrometer were used to record absorption spectra and fluorescence spectrum, respectively. Confocal laser scanning microscopy (Nikon C2+) was used to observe the fluorescence of cells. H&E staining images and Live-Dead images were measured by using Olympus IX73 microscope.

Synthesis

Synthesis of 5-(chlorosulfonyl)-2-(6-(diethylamino)-3-(diethyliminio)-3H-xanthen-9-yl) benzene sulfonate (compound 3) [1]: Sulforhodamine B sodium salt (580 mg, 1 mmol) was dissolved in anhydrous dichloromethane (25 mL) at 0 °C in nitrogen atmosphere. Then, oxalyl chloride (430 μL, 5 mmol) and catalytic amount anhydrous DMF (12 μL) were added slowly under stirring. The resulting mixture was continued

to react for 16 hours at room temperature. The solvent was concentrated in vacuum, dried, and proceeded directly to the next reaction. Yield: 98.0%.

Synthesis of 5-(N-(2-((tert-butoxycarbonyl) amino) ethyl) sulfamoyl)-2-(6-(diethylamino)-3-(diethyliminio)-3H-xanthen-9-yl) benzenesulfonate (4) [1]*:* Compound 3 was dissolved in anhydrous dichloromethane (20 mL) at 0 °C in nitrogen atmosphere. Then, *N*-Boc-ethylenediamine (160 mg, 1 mmol), DMAP (6.1 mg, 0.05 mmol), and triethylamine (420 μL, 3 mmol) were dissolved in dichloromethane (20 mL), and the mixed solution was added dropwise into a solution of Compound 1. The reaction continued for 13 hours at room temperature. After the end of the reaction, the mixed solution was washed with 5 % HCl (50 mL), water (50 mL), and dried over $NaSO_4$. The organic phase was concentrated and the crude product was purified by silica gel column chromatography using CH_2Cl_2:CH_3OH (50:1, v/v) as eluent to obtain a purple solid Compound 4. Yield: 46.0%. ^{1}H NMR (400 MHz, DMSO-d6) δ (ppm): 8.40 (s, 1H), 8.03 (t, J = 6 Hz, 1H), 7.91 (d, J = 8.0 Hz, 1H), 7.46 (d, J = 8.0 Hz, 1H), 7.05-6.93 (m, 6H), 6.87 (t, J = 5.6 Hz, 1H), 3.64 (q, 8H), 3.01 (q, 2H), 2.87 (q, 2H), 1.37 (s, 9H), 1.21 (t, J = 6.8 Hz, 12 H).

Synthesis of 5-(N-(2-aminoethyl) sulfamoyl)-2-(6-(diethylamino)-3-(diethyliminio)-3H-xanthen-9-yl) benzenesulfonate (5) [1]*:* Compound 4 (300 mg, 0.43 mmol) was dissolved in dichloromethane (30 mL). Then, trifluoroacetic acid (3 mL) was added slowly and the reaction was stirred for 3 hours at 0 °C. After the end of the reaction, the solvent and residual trifluoroacetic acid were removed by concentration under vacuum. The crude product was dissolved in dichloromethane (60 mL) and washed twice with saturated sodium carbonate solution (30 mL). The organic solvent was dried over $NaSO_4$ and concentrated to give a red solid compound 5 (RhB-NH2). Yield: 63.4%. ^{1}H NMR (400 MHz, CDCl3) δ (ppm): 8.42 (s, 1H), 7.93 (d, J = 8.0 Hz, 1H), 7.47 (d, J =

8.0 Hz, 1H), 7.06-6.93 (m, 6H), 3.65 (q, 8H), 2.88 (t, *J* = 6.4 Hz, 2H), 2.64 (t, *J* = 6.4 Hz, 2H), 1.21 (t, *J* = 6.8 Hz, 12 H).

Synthesis of 2,2'-disulfanediyldiacetyl chloride (1) [2]*:* Dithiodiglycolic Acid (182 mg, 1 mmol) was dissolved in anhydrous THF (10 mL) at 0 °C in nitrogen atmosphere. Then, oxalyl chloride (105 µL, 1.2 mmol) was added dropwise to the above solution. The mixture continued to react at 35 °C for 3 hours. The solvent and oxalyl chloride were removed by concentration under vacuum to obtain a yellow oil as compound 1, which was used for the next step without further purification.

Synthesis of CPT-SS-COOH (2) [3]*:* CPT (90 mg, 0.26 mmol) and compound 1 were dissolved in anhydrous dichloromethane (20 mL) at 0 °C in nitrogen atmosphere. Then, 4-Dimethylaminopyridine (190 mg, 1.56 mmol) was added to the above solution, and the solution was stirred at 0 °C for 4 hours. The mixture was washed with water (50 mL) and dried over $NaSO_4$. The organic phase was concentrated, and the crude product was purified by flash column chromatography using CH_2Cl_2:CH_3OH (50:1, v/v) as eluent to obtain a yellow solid as compound 2. Yield: 60.1%.

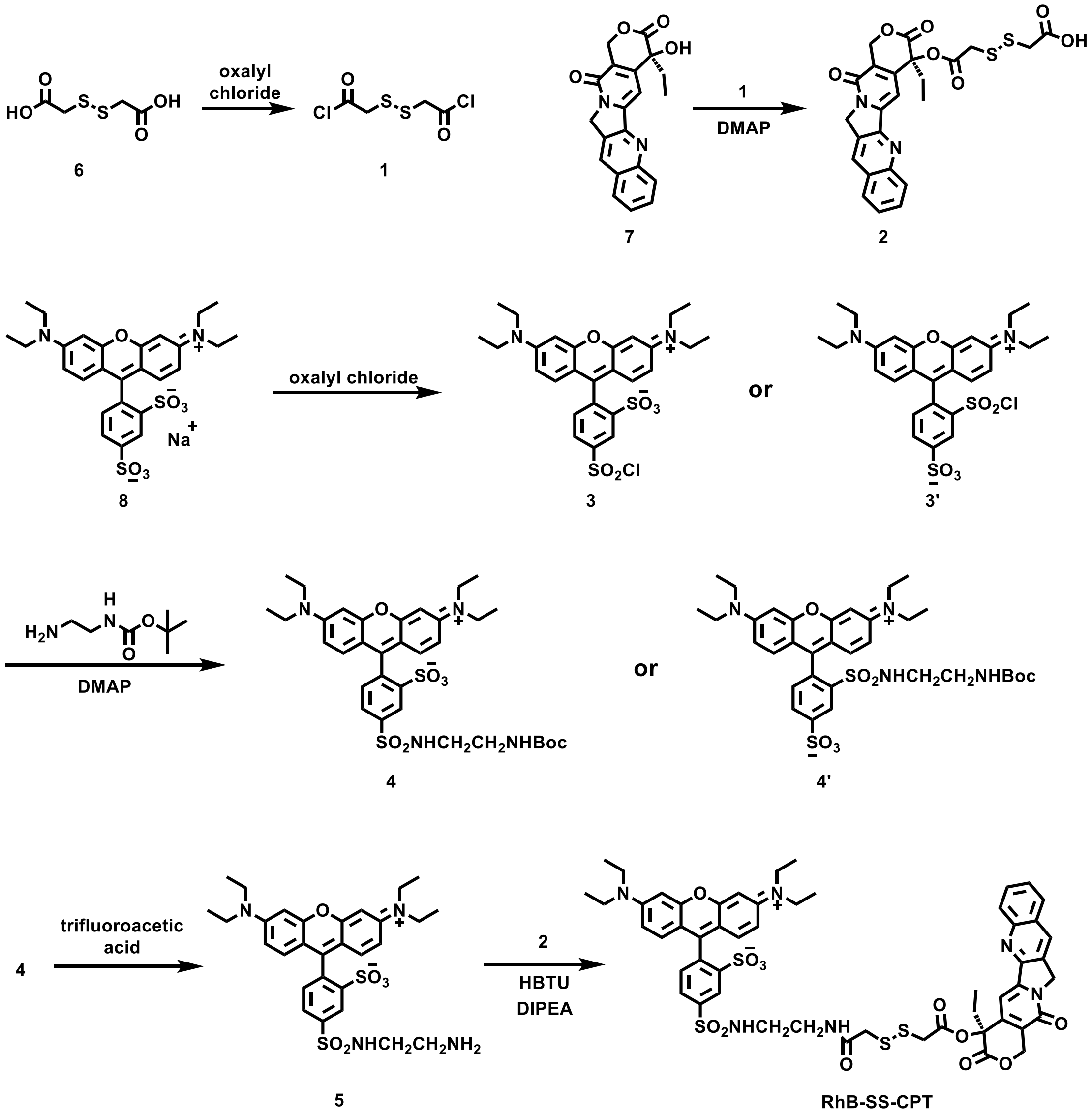

Figure S1. Synthesis route of RhB-SS-CPT.

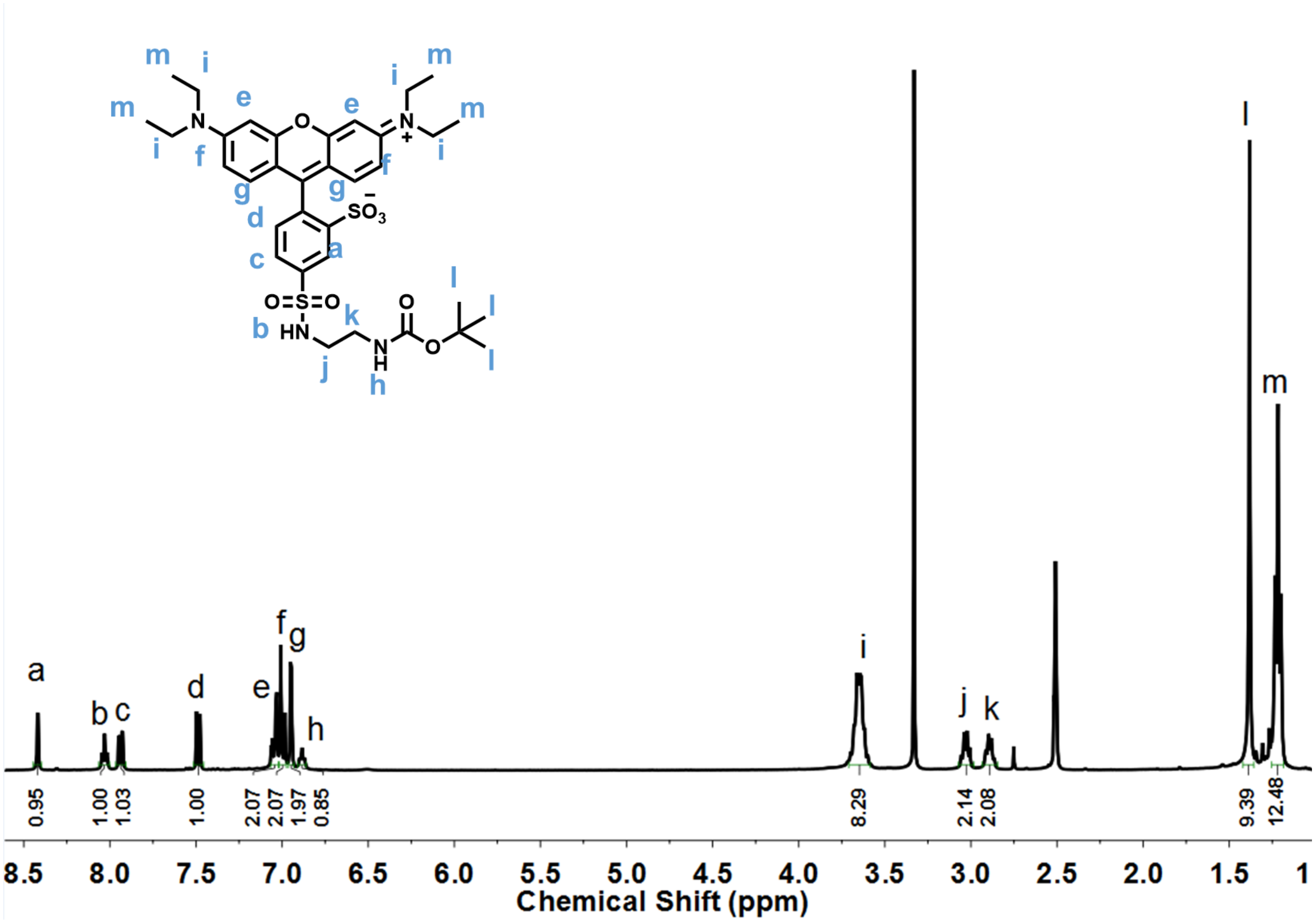

Figure S2. ^{1}H NMR spectrum of compound 4 in DMSO at 298 K.

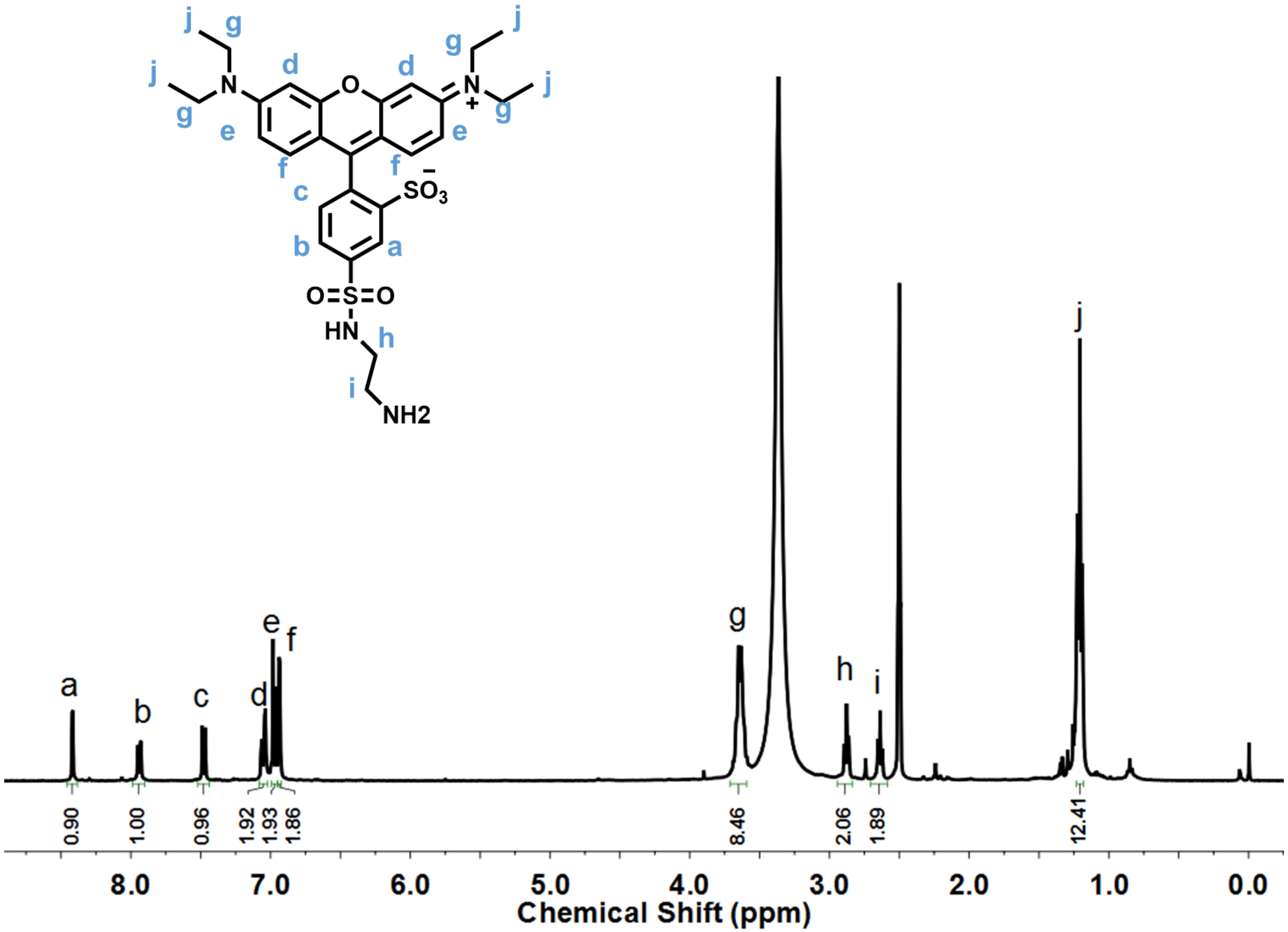

Figure S3. ^{1}H NMR spectrum of compound 5 in DMSO at 298 K.

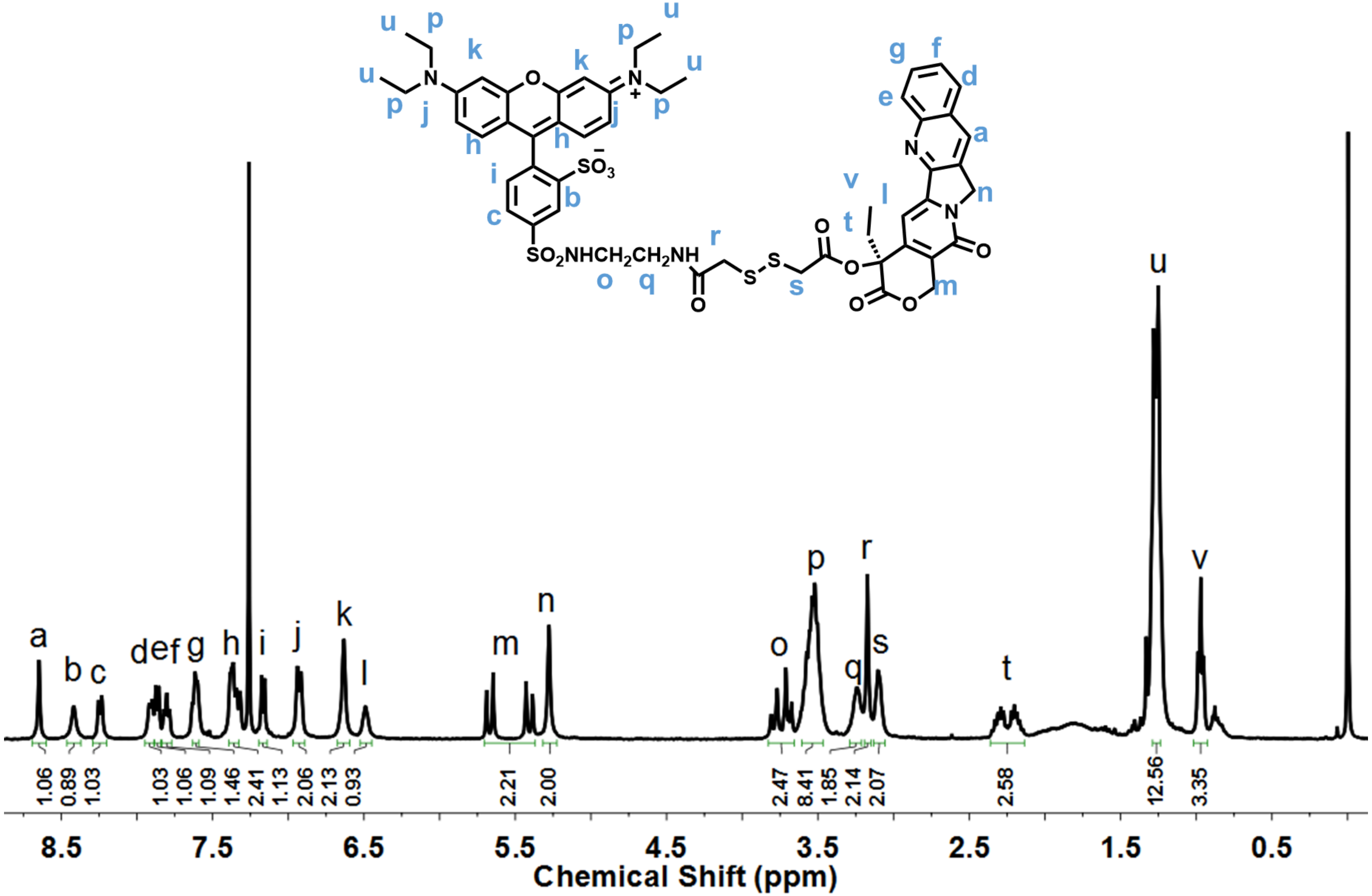

Figure S4. ^{1}H NMR spectrum of RhB-SS-CPT in $CDCl_3$ at 298 K.

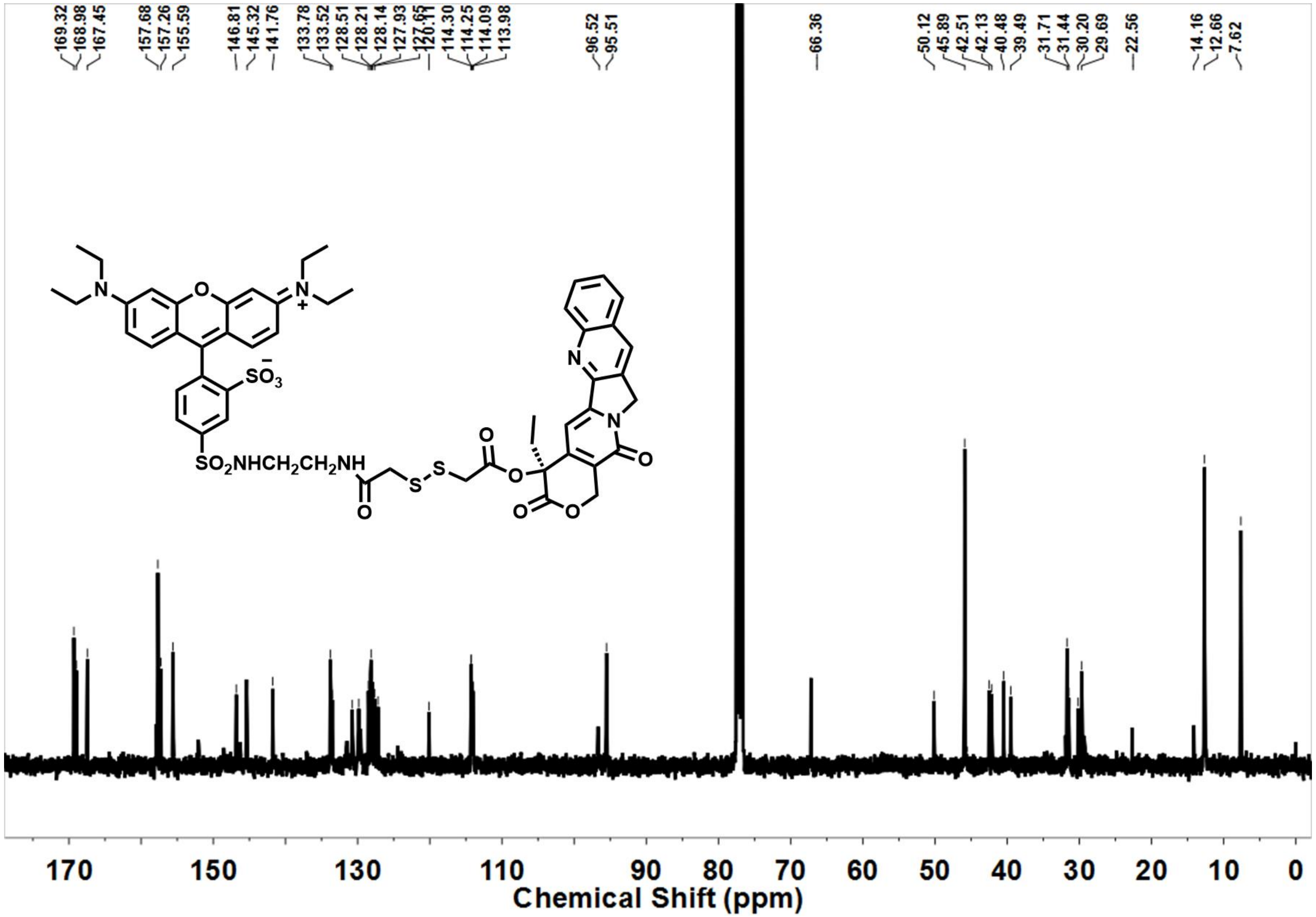

Figure S5. ^{13}C NMR spectrum of RhB-SS-CPT in $CDCl_3$ at 298 K.

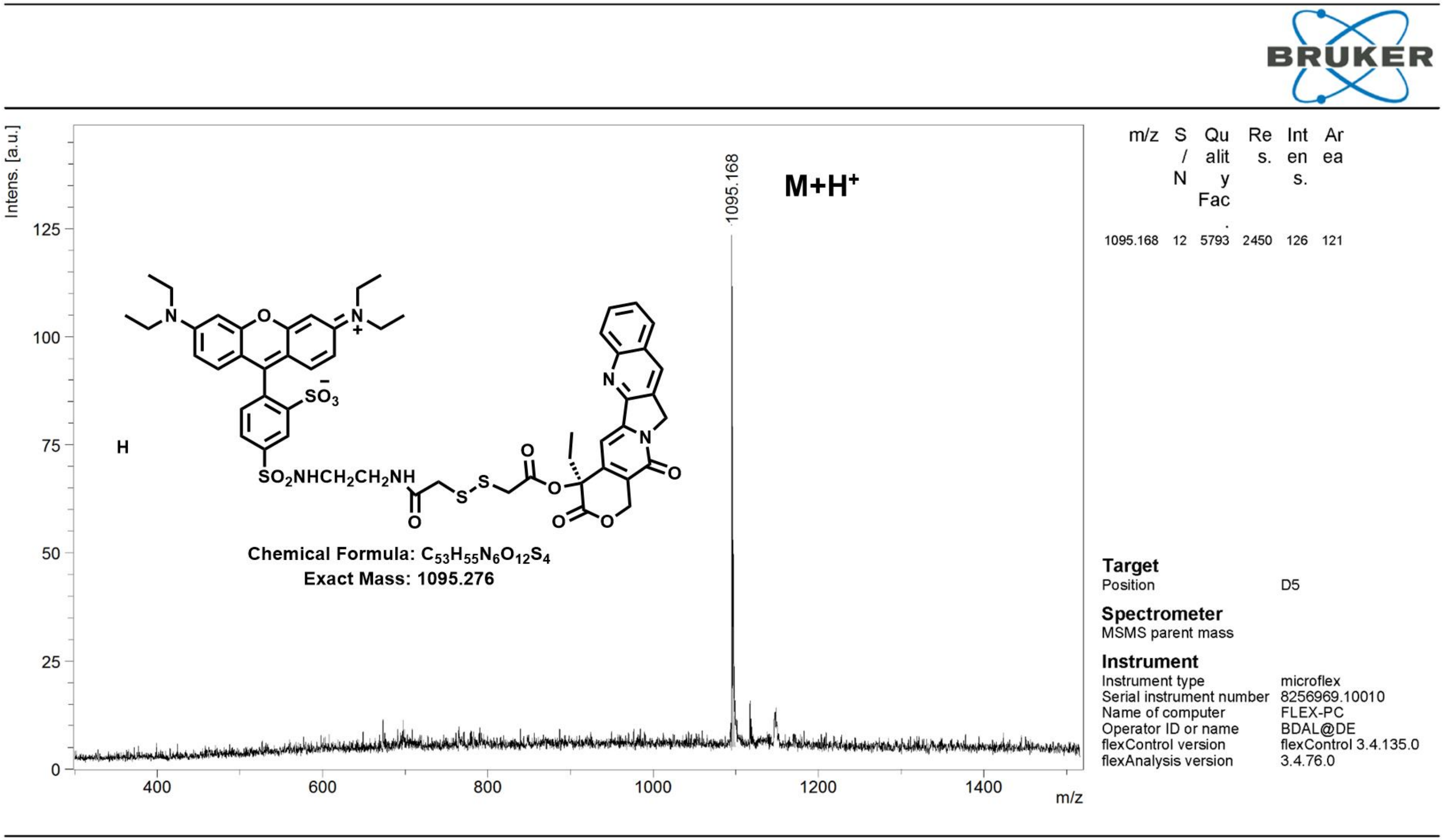

Figure S6. The MALDI-TOF spectrum of RhB-SS-CPT.

Preparation and characterization of RhB-SS-CPT nanoparticles

RhB-SS-CPT prodrug nanoparticles were prepared according to previous literature reports with some modifications.[4] The dimethyl sulfoxide (DMSO) solution of RhB-SS-CPT was slowly added to deionized water and stirred for 30 min with different concentrations, and then the RhB-SS-CPT nanoparticles were prepared. The amount of DMSO can be controlled below 0.5% and can be removed by dialysis.

The size distribution of RhB-SS-CPT prodrug nanoparticles was measured with a Zetasizer 3000 HS (Malvern Instruments, U.K.) at 25 °C. The morphology of prodrug nanoparticles was obtained by using JEM-2100 transmission electron microscope (TEM, JEOL, Japan). The stability of the RhB-SS-CPT prodrug nanoparticles was evaluated by incubating the nanoparticles in PBS containing 10% of fetal bovine serum (FBS). At predetermined time points, the UV-Vis and fluorescence spectra of RhB-SS-CPT prodrug nanoparticles were measured.

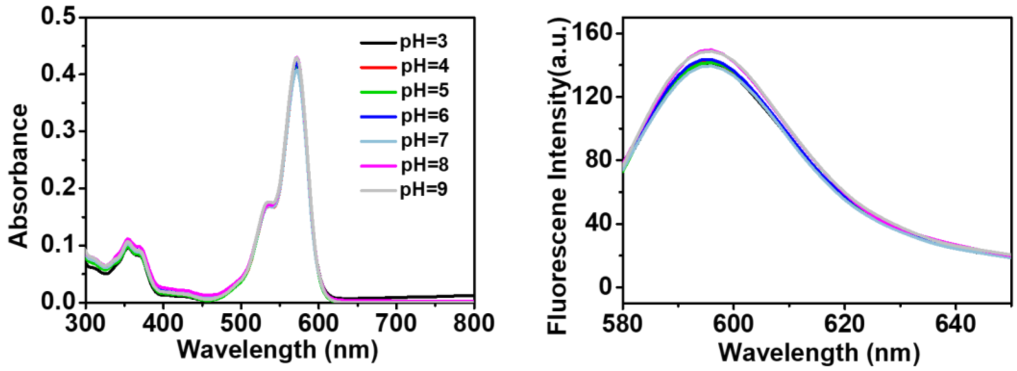

Figure S7. Changes of absorption spectra (Left) and fluorescence spectra (Right) of RhB-SS-CPT in aqueous solution at different pH values.

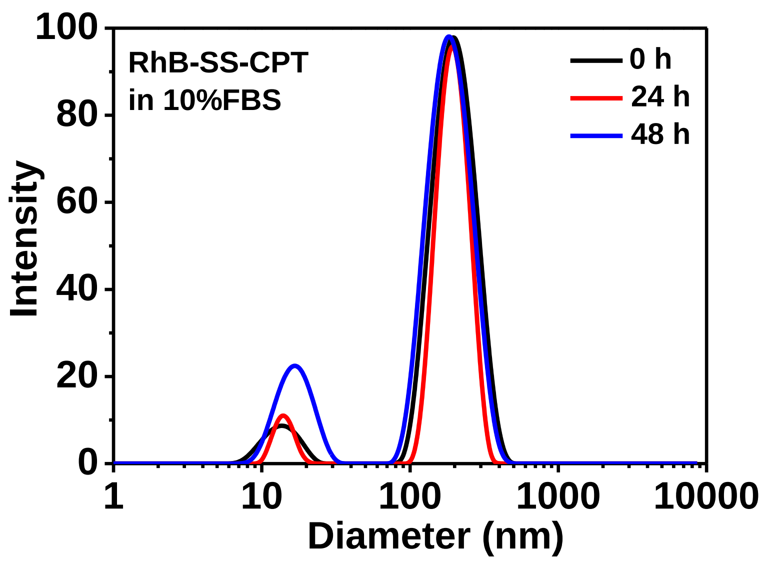

Figure S8. DLS size profiles of RhB-SS-CPT nanoparticles in cell culture medium supplemented with 10% FBS for 0 h, 24 h and 48 h. The concentration of RhB-SS-CPT nanoparticles was 10 μM.

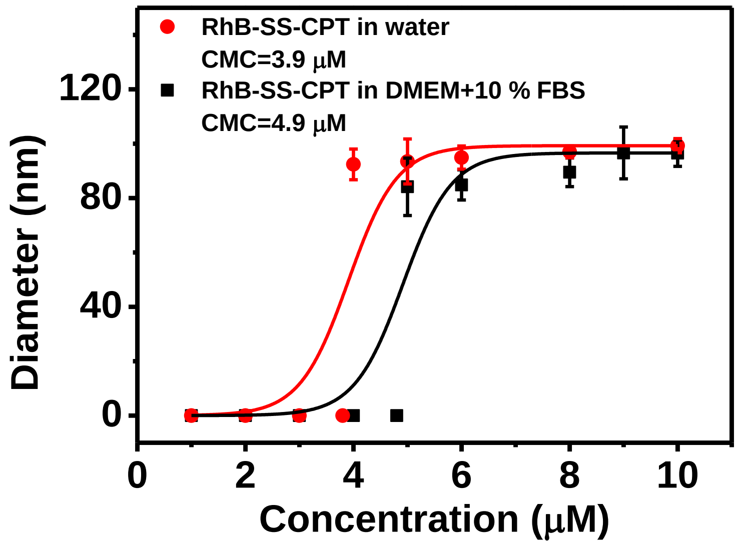

Figure S9. The critical micelle concentration (CMC) of RhB-SS-CPT in water or FBS-supplemented cell medium was assessed by the dynamic light scattering (DLS) method.

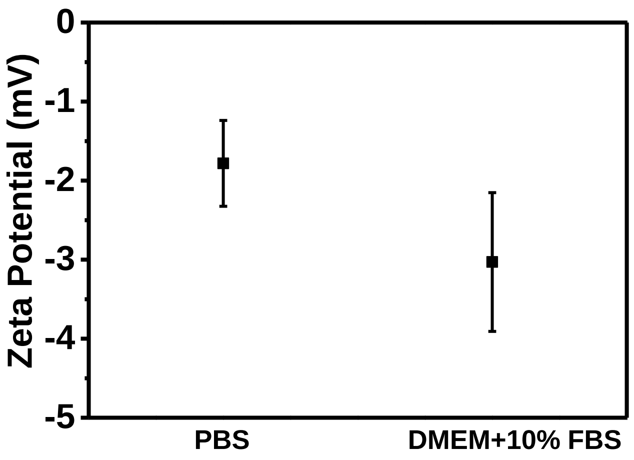

Figure S10. Zeta potential of RhB-SS-CPT nanoparticles in PBS w/o 10% of FBS.

In Vitro CPT release

The release profiles of CPT from prodrug nanoparticles were studied in PBS (pH 7.4, containing 30% ethanol) [5]. RhB-SS-CPT (15 μM) were incubated with different concentrations of H_2O_2 or GSH in 2 mL release media at 37 °C. At predetermined time points, the concentration of the released CPT from prodrug nanoparticles was determined by high performance liquid chromatography (HPLC) (n=3 for each group). The HPLC analysis of drug release was achieved on Agilent InfinityLab Poroshell 120 EC-C18 4 μm (4.6×150 mm) Column with a mobile phase of CH_3OH: H_2O=60:40, a column temperature at 20 °C, a flow rate at 1 mL/min, and a UV detection wavelength at 364 nm. To further investigate the mechanism of H_2O_2-triggered or GSH-triggered CPT release, ESI-MS was used to monitor the molecular weight of RhB-SS-CPT nanoparticles after treated with H_2O_2 or GSH (10 mM) for 6 h.

In Vitro cytotoxicity assay

HeLa and L929 cells were cultured in a conventional medium supplemented with 10% FBS and 1% penicillin/streptomycin. All cells were maintained in a humidified atmosphere of 5% CO_2 at 37 °C. Firstly, HeLa cells were used to evaluate the cellular

uptake of RhB-SS-CPT prodrug nanoparticles. Briefly, cells were seeded in glass bottom dish at a density of 1 × 10^5 cells and incubated for 24 h. Then, cells were washed with PBS and incubated with RhB-SS-CPT nanoparticles (5 μM) for 0.5 h or 2 h at 37 °C. The treated cells were washed with PBS for three times and co-stained with Hoechst 33342 for 5 min. The fluorescence imaging of cells was photographed under confocal laser scanning microscopy (Nikon C2+).

The HeLa and L929 cells were used to evaluate the anticancer activity of RhB-SS-CPT nanoparticle in vitro. The free CPT and saline were used as controls. After incubation for 12 h, the cell medium was removed and replaced with 200 μL of medium containing serially diluted RhB-SS-CPT nanoparticles or free CPT from 0.1 to 100 μM. The cells treated with saline were used as negative control. The cells were incubated at 37 °C with 5% CO_2 for another 72 h. Live/Dead reagent (calcein-AM/EthD-1) were added to each well, and the cells were photographed with fluorescence microscope (Olympus IX73).

In Vivo anticancer efficacy

Firstly, Kunming mice were used to evaluate the pharmacokinetic profiles of RhB-SS-CPT nanoparticles. The animals were randomly divided into free CPT and RhB-SS-CPT groups, and were intravenously administrated with free CPT or RhB-SS-CPT at a dose of 4 mg/kg equivalent to CPT (n = 3 for each group). At predetermined time points, blood samples were collected, and 10 μL of blood was lysed by 50 μL RIPA Lysis buffer. Then 50 μL of DMSO was added, and the mixture was analyzed on Tecan Microplate reader to calculate the concentration of CPT.

Then, 4T1 cells (3 × 10^6 cells per 200 μL) were injected subcutaneously into the right hind leg of female nude mice (6 six weeks old, about 15 g). When the tumor volume reached 250 mm^3, the nude mice were divided into two groups and intravenously

injected with RhB-SS-CPT nanoparticles at a dose of 5 mg/kg equivalent to CPT (n=3 for each group). After 6 h, 12 h, 30 h, or 48 h after injection, mice were sacrificed and hearts, livers, spleens, lungs, kidneys and tumors were collected. PerkinElmer IVIS lumina Series III in vivo fluorescence imaging system was used to record the fluorescence intensity.

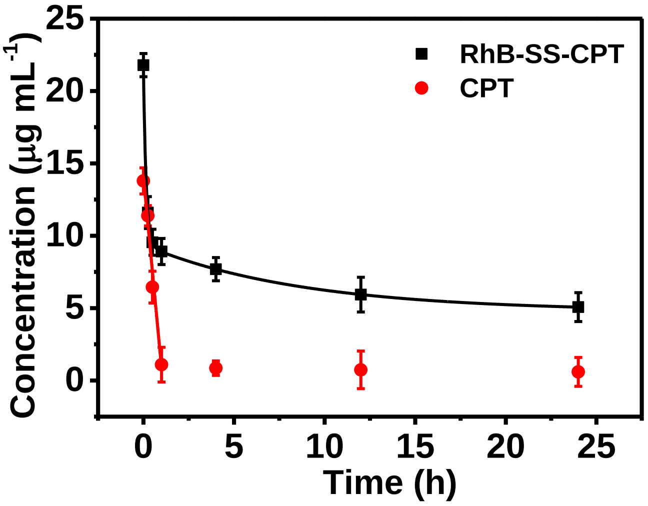

Figure S11. Concentration of CPT in blood at different time points after intravenous injection of RhB-SS-CPT NPs or CPT.

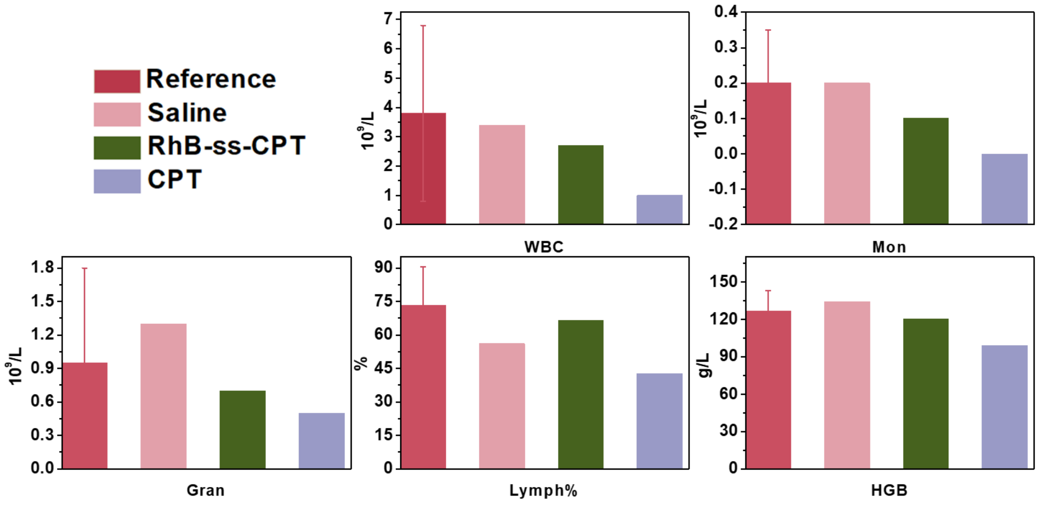

Figure S12. Blood test parameters of the mice treated with saline, CPT, or RhB-SS-CPT. Red bar represents the reference value.

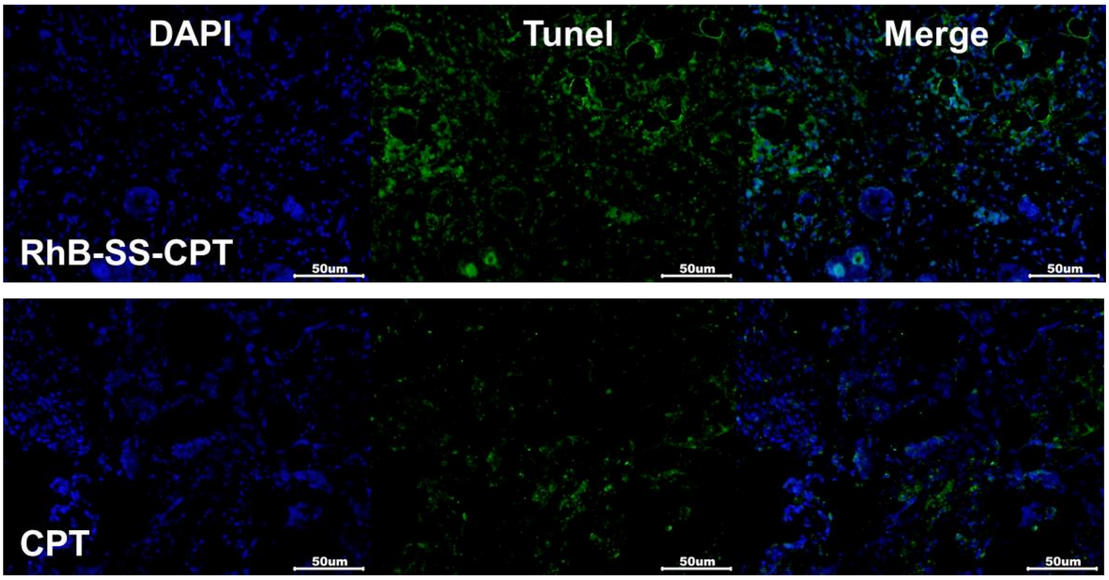

Figure S13. TUNEL staining of tumor tissue sections showing more apoptotic cells in the RhB-SS-CPT treatment group than in the CPT-treated group.

Reference

1. Wang YJ, Liu D, Zheng QC, Zhao Q, Zhang HJ, Ma Y, et al. Disulfide Bond Bridge Insertion Turns Hydrophobic Anticancer Prodrugs into Self-Assembled Nanomedicines. Nano Lett. 2014; 14: 5577-83.
2. Cao Y, Gao M, Chen C, Fan A, Zhang J, Kong D, et al. Triggered-release polymeric conjugate micelles for on-demand intracellular drug delivery. Nanotechnol. 2015; 26: 115101.
3. Phua SZF, Xue CC, Lim WQ, Yang GB, Chen HZ, Zhang YY, et al. Light-Responsive Prodrug-Based Supramolecular Nanosystems for Site-Specific Combination Therapy of Cancer. Chem Mater. 2019; 31: 3349-58.
4. Huang P, Wang DL, Su Y, Huang W, Zhou YF, Cui DX, et al. Combination of Small Molecule Prodrug and Nanodrug Delivery: Amphiphilic Drug-Drug Conjugate for Cancer Therapy. J Am Chem Soc. 2014; 136: 11748-56.
5. Sun BJ, Luo C, Yu H, Zhang XB, Chen Q, Yang WQ, et al. Disulfide Bond-Driven Oxidation- and Reduction-Responsive Prodrug Nanoassemblies for Cancer Therapy. Nano Lett. 2018; 18: 3643-50.
